# Myoelectric Control Performance of Two Degree of Freedom Hand-Wrist Prosthesis by Able-Bodied and Limb-Absent Subjects

**DOI:** 10.1109/TNSRE.2022.3163149

**Published:** 2022-04-11

**Authors:** Ziling Zhu, Jianan Li, William J. Boyd, Carlos Martinez-Luna, Chenyun Dai, Haopeng Wang, He Wang, Xinming Huang, Todd R. Farrell, Edward A. Clancy

**Affiliations:** Department of Electrical and Computer Engineering, Worcester Polytechnic Institute, Worcester, MA 01609 USA; Department of Electrical and Computer Engineering, Worcester Polytechnic Institute, Worcester, MA 01609 USA; Department of Electrical and Computer Engineering, Worcester Polytechnic Institute, Worcester, MA 01609 USA; Liberating Technologies, Inc., Holliston, MA 01746 USA; Department of Electrical Engineering, Fudan University, Shanghai 200433, China; Department of Electrical and Computer Engineering, Worcester Polytechnic Institute, Worcester, MA 01609 USA; Department of Electrical and Computer Engineering, Worcester Polytechnic Institute, Worcester, MA 01609 USA; Department of Electrical and Computer Engineering, Worcester Polytechnic Institute, Worcester, MA 01609 USA; Liberating Technologies, Inc., Holliston, MA 01746 USA; Department of Electrical and Computer Engineering, Worcester Polytechnic Institute, Worcester, MA 01609 USA

**Keywords:** Prosthesis control, EMG-force, EMG signal processing, electromyogram, myoelectric control

## Abstract

Recent research has advanced two degree-of-freedom (DoF), simultaneous, independent and proportional control of hand-wrist prostheses using surface electromyogram signals from remnant muscles as the control input. We evaluated two such regression-based controllers, along with conventional, sequential two-site control with co-contraction mode switching (SeqCon), in box-block, refined-clothespin and door-knob tasks, on 10 able-bodied and 4 limb-absent subjects. Subjects operated a commercial hand and wrist using a socket bypass harness. One 2-DoF controller (DirCon) related the intuitive hand actions of open-close and pronation-supination to the associated prosthesis hand-wrist actions, respectively. The other (MapCon) mapped myoelectrically more distinct, but less intuitive, actions of wrist flexion-extension and ulnar-radial deviation. Each 2-DoF controller was calibrated from separate 90 s calibration contractions. SeqCon performed better statistically than MapCon in the predominantly 1-DoF box-block task (> 20 blocks/minute vs. 8–18 blocks/minute, on average). In this task, SeqCon likely benefited from an ability to easily focus on 1-DoF and not inadvertently trigger co-contraction for mode switching. The remaining two tasks require 2-DoFs, and both 2-DoF controllers each performed better (factor of 2–4) than SeqCon. We also compared the use of 12 vs. 6 optimally-selected EMG electrodes as inputs, finding no statistical difference. Overall, we provide further evidence of the benefits of regression-based EMG prosthesis control of 2-DoFs in the hand-wrist.

## I. Introduction

More than two million people live with limb absence in the U.S., and this number increases by an average 185,000 each year [1]–[3]. Trans-radial amputations make up 60% of total wrist and hand amputations, and documented rates of prosthesis use vary from 27–56% for upper-limb amputation [4]. The high demand for prostheses, expected to increase by at least 47% by the year 2020, has brought more support from government and growth of the market [5].

While laboratory-based research on electromyogram (EMG) control has generated new strategies based on machine learning algorithms, most commercial prostheses still use simple two-site control schemes that have been available for decades [6]. Typical myoelectric prosthesis sockets are designed with two bipolar electrodes, one each located over extensor and flexor muscles, to control one degree-of-freedom (DoF) prosthesis hand open and close (Opn-Cls), respectively. Kestner [7] found need for a prosthetic wrist, as the fixed angle of a prosthetic hand is not compatible with all daily tasks (e.g., holding flatware for eating, a bottle for drinking). Although some advanced prostheses have a wrist rotator and users can co-contract their muscles to switch between hand open-close and wrist pronation-supination (Pro-Sup) [8], [9], users mostly employ their body and arm/shoulder movement for compensation instead [10]–[12]. Prosthesis mode switching, a.k.a. sequential 2-DoF control via co-contraction mode switching, allows users to rotate the wrist with a complex and time-consuming approach [13]. Performance of this technique is highly influenced by a user’s residual limb condition, since muscle contraction imbalance or neuron damage impede co-contraction; and users need a long period of time to master this skill, but easily fatigue [14].

Features extracted from myoelectric signals train models to estimate users’ intent. Regression modeling is one learning approach used to realize simultaneous, independent and proportional multi-DoF control [15]–[19]. Compared with classification models, of which numerous varieties have been investigated [20]–[25], the continuous outputs of regression estimates may more naturally mimic human movement. Regression models have been found to be more robust to some unpredictable small variations in EMG signals, such as fatigue or poor contact of electrodes, and may generate better performance during untrained conditions compared to classification models [19].

Most upper-limb myoelectric control users can easily operate hand open-close via the two-site conventional approach. But for wrist rotation—although most limb-absent users can easily rotate their residual limb repeatedly—the supinator (a wrist rotator) is a deep muscle difficult to record using surface EMG [26], and electrodes often shift during forearm rotation. These factors challenge the usability of surface EMG signals. As an alternative, researchers assessed offline other wrist motions of extension-flexion (Ext-Flx) and radial-ulnar deviation (Rad-Uln), especially since the EMG signal during Rad-Uln has demonstrably distinct patterns compared with the other wrist motions [27]. In the context of proportional control of multiple DoFs, “distinct” patterns are most clearly demonstrated when unique EMG channels record large amplitude EMG when contracting directly along one motion (e.g., radial deviation) and low amplitude EMG when contracting directly along all other motions. These results provide a potential 2-DoF control strategy by a corresponding “motion” mapping/translation.

Some prior lab-based prostheses testing of multiple-DoF control schemes used a large number of electrodes, or matrix electrodes. Such systems are not practical in current commercial prostheses due to cost and issues of electrode shorting/lift-off. Some researchers found that at least 4 electrodes were necessary to realize 2-DoF control, with improvement occurring if the number of electrodes increased [28]–[30]. A balance can be found between economic benefits and product quality if an optimal number of electrodes and their location were decided [28], [31].

Recent laboratory work studied myoelectric control using a 2-D virtual target tracing task, assessing performance via path efficiency, completion time, and attempt-ratio [32]. Others have studied the influence of training protocol [33], or of using modeling techniques of myoelectric representation learning (MRL) [34], principle component analysis (PCA) [33], and frequency division technique (FDT) [35]. Real 2-DoF prosthesis control during either laboratory or home study found a potential advantage of regression-based controllers [36] and classification-based [37], [38] in multi-DoF control compared with conventional control strategies.

Different regression-based approaches have been evaluated offline and online (e.g., [18], [32], [34]–[36], [39]). Most studies have used commercial prosthesis hardware, with custom controllers. Electrode site selection is usually circumferential around the forearm (for hand-wrist prosthesis) with equal inter-electrode distances, or manually selected based on residual anatomy. The number of electrodes used has varied. A few studies have combined pattern recognition with proportional control [40], [41] (as have some commercial products). A fundamental limitation of all of this work is the limited sample size (often ≤10), which seems necessitated by the complexity and cost of such studies [42], [43]. The aggregate sample size of limb-absent subjects that have tested such systems is even smaller (as small as one limb-absent subject in some studies). Hence, there exists no standard approach to regression-based multi-DoF simultaneous, proportional control of prostheses, particularly in controller calibration, regression method, number of electrodes used, electrode site selection, etc.; nor have its advantages vs. disadvantages with respect to other control approaches been adequately understood.

In this paper, we assessed regression-based simultaneous, independent and proportional 2-DoF (hand-wrist) myoelectric prosthesis control on both able-bodied and limb-absent subjects, comparing three control strategies—Opn-Cls & Pro-Sup direct control, a new Ext-Flx & Rad-Uln mapping control with translation, and conventional two-site sequential control. Six or twelve optimally-sited electrodes (out of 16 total) were tested on a prosthesis to investigate the minimum number of electrodes feasible on commercial prostheses. Bypass brackets were designed separately for able-bodied and limb-absent subjects to carry a hand-wrist prosthesis adjacent to the forearm/residual limb. Each bypass allowed subjects to don the prosthesis without a socket, while allowing access to the limb for electrode placement. The three control strategies were tested with different standard physical tasks—box-block, refined-clothespin relocation and door-knob (the latter two requiring use of 2-DoFs). Six vs. twelve optimally-selected electrodes were tested to explore the minimum number of necessary electrodes for able-bodied subjects. Based on these results, more targeted tasks were conducted on limb-absent subjects.

## II. Methods

### A. Experimental Apparatus

Experimental data were collected from 10 able-bodied (5 male, 5 female; aged 18–45 years) and 4 trans-radial limb-absent (3 male, 1 female; aged 39–65 years; 2 congenital, 2 traumatic amputee) subjects at Worcester Polytechnic Institute (WPI), as approved by the WPI Institutional Review Board (IRB Protocol 17–155). Able-bodied subjects had no physical limitations of their dominant forearm muscles. Limb-absent subjects had ≥5 cm residual limb length with functional muscle contraction and prior experience with myoelectric-controlled prostheses. Subjects provided written informed consent.

**Fig. 1.**
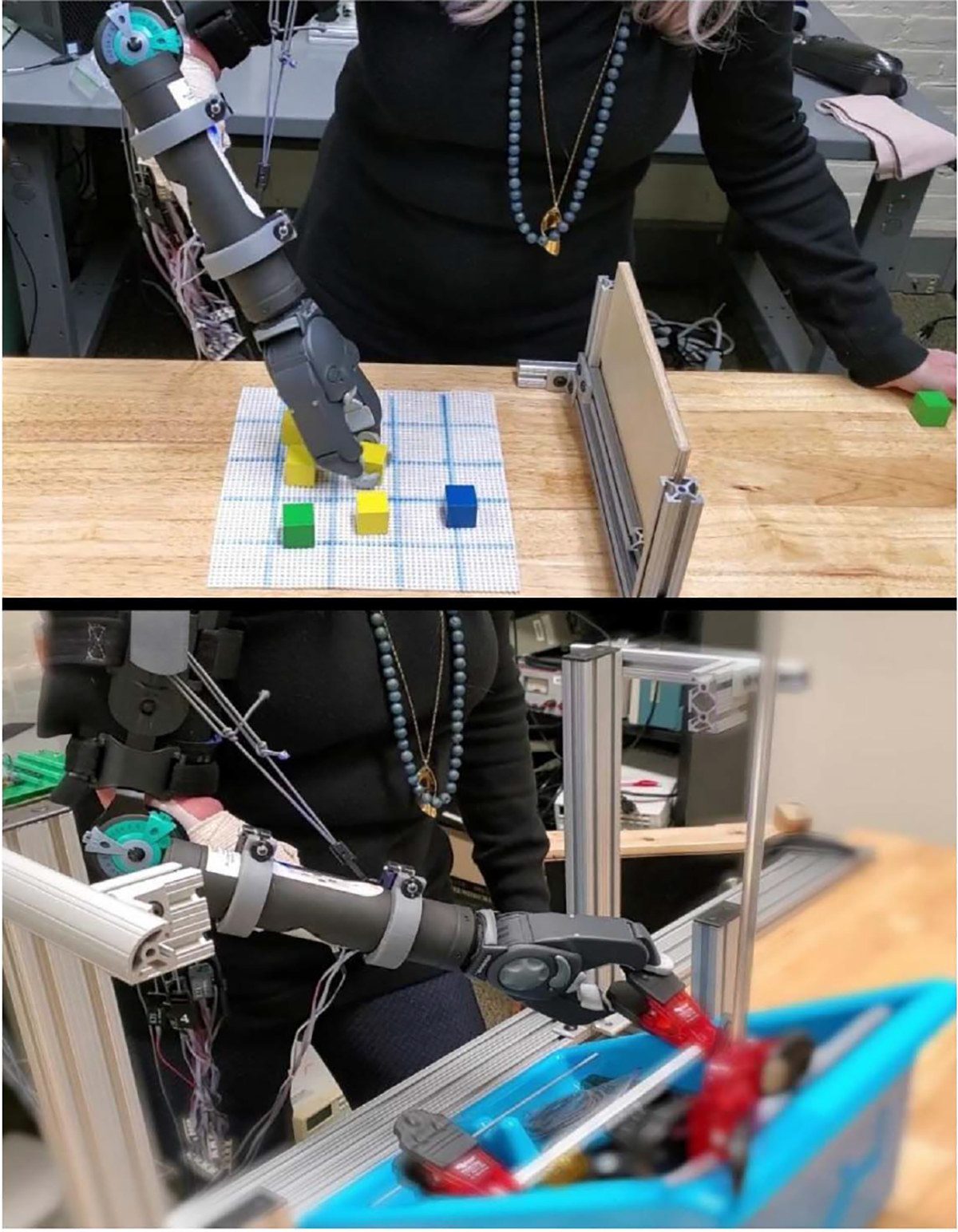
Experimental apparatus for box-block (top) and clothes pin (bottom) tasks, limb-absent subject. The subject was asked to wear a bypass bracket that attached a hand-wrist prosthesis. The forearm could move freely.

Subjects stood at the experimental table, adjusted to hip height (Fig. 1). Sixteen bipolar EMG electrodes were secured on the proximal forearm, equally spaced about the forearm’s circumference. For able-bodied subjects, electrodes were secured on the dominant side with the midpoint of the bipolar contacts placed 5 cm distal to the elbow crease. For limb-absent subjects, electrodes were secured on the affected side at the level corresponding to that of their own prosthesis. Each bipolar electrode consisted of 5 mm diameter, stainless steel, hemispherical contacts separated 1 cm edge-to-edge, oriented along the forearm’s long axis. Each EMG signal was differentially amplified (Liberating Technologies, Inc. BE328 amplifier; 30–500 Hz pass band, CMRR > 100 dB over the pass band) and provided selectable gain. All EMG channels were sampled at 2000 Hz on a PC (16-bit resolution).

Then a 3D printed bypass prosthesis bracket was strapped to the shoulder and arm on the same side as the electrodes (Fig. 1). A wrist rotator (Fillauer Motion Control Standard Wrist Rotator, maximum speed 28 rpm) and prosthetic terminal device (System Electric Greifer DMC Plus, proportional speed 8–200 mm/sec) extended from the bypass, providing wrist Pro-Sup and hand Opn-Cls, respectively. The electrodes (input) and the prosthesis control signals (output) were part of a PC-based system programed in MATLAB (The MathWorks, Natick, MA, USA) [44]. The main processing loop of this system operated at 100 Hz so as to minimize controller delays.

### B. Prostheses Control System

#### 1) Control Sources:

Subjects compared two regression-based 2-DoF simultaneous, independent and proportional velocity control algorithms, and conventional two-site velocity control. Limb-absent subjects controlled the prostheses by attempting to move their phantom limb. The control algorithms were as follows. 1) Direct control (DirCon) in which subjects’ Opn-Cls controlled Greifer Opn-Cls, and subjects’ Pro-Sup controlled prosthetic wrist rotation. This 2-DoF approach is the most intuitive. 2) Direct control with mapping/translation (MapCon) in which subjects’ wrist Ext-Flx controlled Greifer Opn-Cls (Ext corresponded to Opn), and subjects’ Rad-Uln controlled prosthetic wrist rotation (Rad corresponded to pronation). Subjects were permitted to invert either/both of these mappings (although none chose to do so). 3) Sequential control (SeqCon) in which subjects controlled either Opn-Cls or Pro-Sup, then switched between them by triggering a co-contraction EMG signal. Co-contraction was defined as a simultaneous contraction of both processed forearm EMGs (processing described below) above set thresholds for a defined time duration [45], [46]. Each respective threshold was set between the EMG values triggered during a maximum co-contraction and normal hand-wrist tasks, as selected by the subject. The time duration was set between 30–100 ms, again selected by subject preference.

#### 2) Control Calibration and Thresholding:

**Fig. 2.**
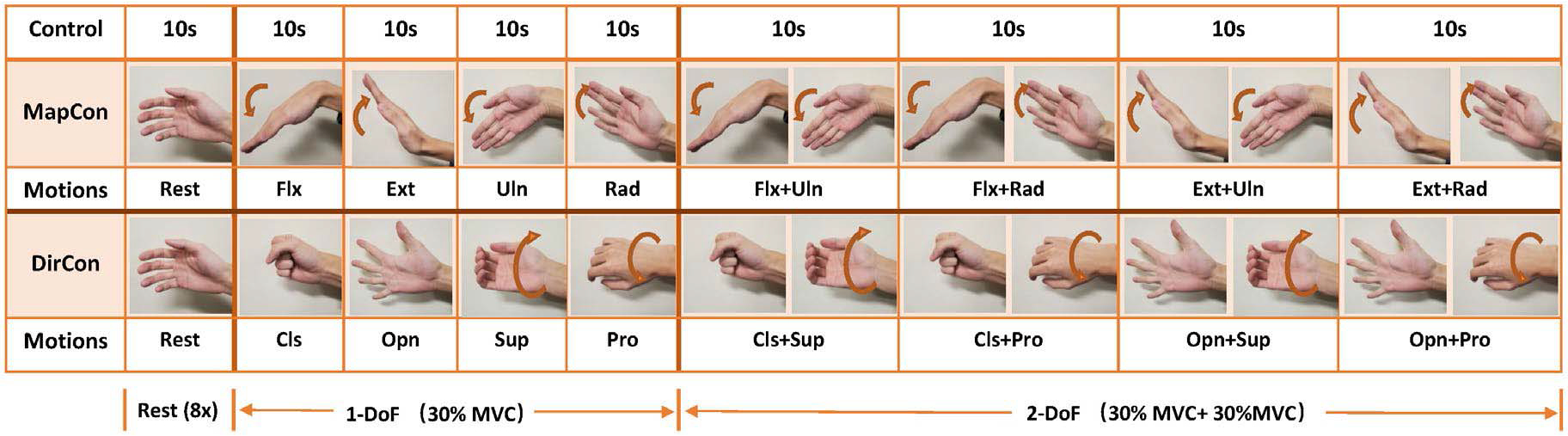
Sequence of calibration contractions. Subjects follow the instructions to perform indicated constant-pose, constant-force contractions over 90 s. The recording was used for coefficient calculation and calibration quality assessment.

For calibration of DirCon and MapCon (Fig. 2), subjects performed a 90 s calibration consisting of 10-s of rest and eight distinct 10-s, contiguous constant-posture constant-force contractions (four 1-DoF and four 2-DoF). Since maximum voluntary contraction (MVC) cannot be measured on the affected side of prosthesis users, all subjects were instructed to maintain, as best as possible, a contraction target effort of 30%—without feedback. MVC was not measured in either the able-bodied or limb-absent subjects. For DirCon, the contraction sequence was: Cls, Opn, Sup, Pro, Cls+Sup, Cls+Pro, Opn+Sup, and Opn+Pro. For MapCon, the contraction sequence was: Flx, Ext, Uln, Rad, Flx+Uln, Flx+Rad, Ext+Uln, and Ext+Rad. Raw EMG signals from all channels were digitally notch filtered (second-order IIR filter at 60 Hz, notch bandwidth of 1 Hz), highpass filtered to attenuate motion artifact (*f_c_* = 15 Hz, fifth-order Butterworth filter), rectified, lowpass filtered (*f_c_* = 16 Hz; Chebyshev Type I filter, ninth-order, 0.05 dB peak-to-peak passband ripple) and downsampled from 2000 Hz to 100 Hz. Then, a critically damped lowpass filter (*f_c_* = 1 Hz, second-order) [47] was applied to further smooth the signal and estimate EMG standard deviation (EMGσ, a.k.a. processed EMG). The first and last second of each 10 s contraction was removed to avoid filter and movement transients. Then, each EMGσ from the resting contraction (weighted eight times) and the eight active contractions were used as inputs to a regression-based (2-output) static EMGσ-force model [31]. Re-using one rest contraction balances the weight of the regression fit, without extending its duration. A force of zero was assigned as the output target for unused DoFs during each contraction. Fit coefficients were estimated via the linear least squares pseudo-inverse method, in which singular values of the design matrix were removed if the ratio of that singular value to the largest was less than a tolerance value (*Tol* = 0.01, based on previous study) [16], [48]. Backward stepwise selection was utilized for optimal selection of either 6 or 12 electrodes (out of 16 total). In this manner, only the best channels yielding the lowest RMSE between EMG-force and target force were used [28], [31], and their gains were calculated for prostheses control. In addition, this RMSE provided an assessment of the calibration quality.

**Fig. 3.**
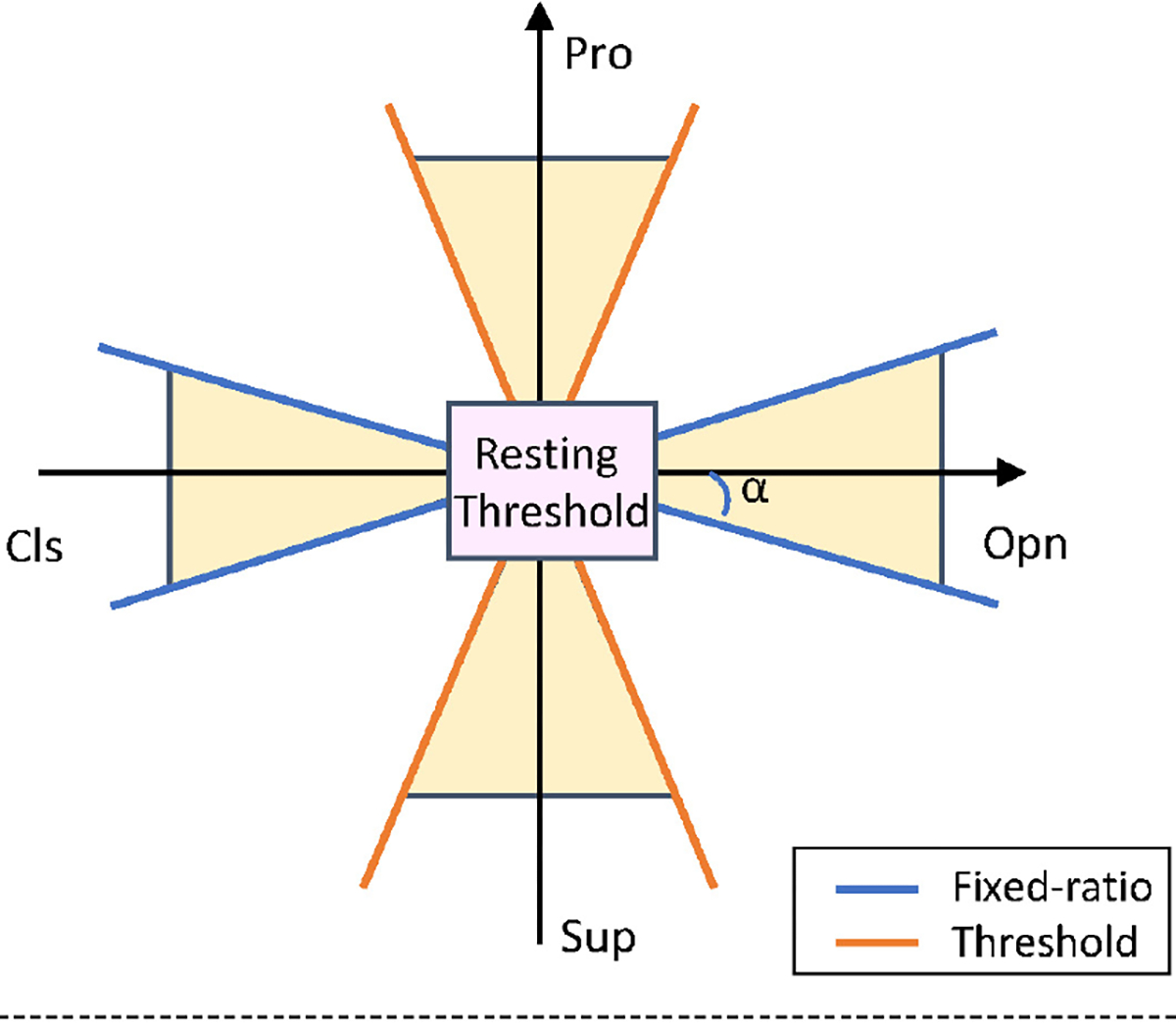
Thresholding methods for 2-DoF control including resting (inner square) and fixed-ratio thresholding (blue and red lines emanating from inner square). Based on method of Fougner *et al*. [49].

During experimental trials using DirCon and MapCon, EMG-force was computed in real-time, then two thresholding methods were applied. First, a resting threshold was applied to each direction of the two individual DoFs (total of four thresholds) to minimize the impact from noise and unintentional EMGσ signals. Initially, the threshold was set to 10 %MVC for each direction. Then, subjects were asked to rest and to slowly move their arm. If unintentional prosthesis movement resulted, the corresponding threshold was slightly increased until no movement occurred. Second, a fixed-ratio co-activation thresholding method was applied to attenuate the risk of inadvertent activation of another DoF (Fig. 3). When the ratio of the larger force (in %MVC) to the smaller force (from the two DoFs) was less than a threshold, only the DoF with the larger force was actuated. If the two forces are drawn in the *x*-*y* plane, a default threshold angle of *α* = 25 degrees [49] was used. This angle could be changed during setup as desired by the subject.

For SeqCon, the two channels which produced the most distinct EMGσ (based on channel amplitudes) when subjects performed Ext and Flx calibration, respectively, were manually chosen. For limb-absent subjects, we selected EMG sites near the location of the sites used by their existing two-site prosthesis controller, whenever multiple distinct channel options existed. Each channel gain was set to correspond to 30% MVC. The estimated force was calculated as the algebraic difference of the forces estimated by each channel. A resting threshold was applied to each channel to reduce the influence of noise and small unintentional activation. For switching between the 2 DoFs, a fixed window size (30–100 ms) and a co-contraction threshold were set to detect a co-contraction. All the channels and coefficients were manually calibrated until subjects could easily control the prostheses and trigger co-contraction.

#### 3) Hardware Control:

The estimated hand and wrist force levels, in %MVC, were linearly mapped to hand and wrist velocity (speed and direction), with 50% MVC in each corresponding to maximum speed. Built-in hardware thresholds were essentially disabled by matching the software thresholds to them. Thus, all thresholding was set in our custom software.

### C. Experimental Protocol

Subjects stood for all tasks, but otherwise their posture was not constrained (Fig. 1). To prevent cumulative muscle fatigue, at least two minutes rest after calibration and one minute rest between trials were provided. All limb-absent subjects completed 10–20 minutes of mirror-box training before the trials to help rebuild their phantom limb control sensation. The two traumatic amputees had prior mirror-box training experience.

To assess controller performance, three tasks were chosen from widely-used outcome measures described in the literature. 1) The box-block task [50] was a 1-DoF assessment mainly testing hand Opn-Cls. Subjects grasp (hand Cls) a block and then drop it (hand Opn) after traversing over a partition. They return back over the partition and repeat. We did not lock the prostheses into 1-DoF control during this task. The number of transferred blocks in 60 s and number of drops were measured in each trial. 2) The refined-clothespin relocation task [51] was a 2-DoF assessment. Subjects perform hand Cls to grasp a clothespin (2 lbs. resistance) from a horizontal rod, rotate the clothespin 90° (wrist Pro or Sup), then place and release (hand Opn) the clothespin onto a vertical rod. Once complete, subjects rotate their wrist back to its original orientation and attempt to relocate another clothespin. Subjects were allowed to use arm or body movement for compensation. If the clothespin dropped, subjects moved on to the next clothespin. The time required to complete three successful moves (maximum of 120 s) and number of drops were measured in each trial. 3) The doorknob task was a 2-DoF assessment. Opening a door is a common but important task that most people face every day. Compared with the SHAP door-handle test [52], our task used a round knob so as to require actuation of both the wrist and hand—more appropriate for 2-DoF assessment. During each task cycle, subjects grasped the round knob of the door (hand Cls), rotated the knob (wrist Pro or Sup), pulled the door open, and then released the knob (hand Opn). Subjects then shut the door to ready for the next trial. The time required to complete three successful door openings (maximum of 120 s) was measured in each trial.

Three control strategies (DirCon, MapCon, SeqCon) were tested on all subjects. Subjects initially performed calibration, then used all 16 electrodes to test all motions and their combinations. Thresholds were adjusted, based on their feedback, to enhance control robustness and accuracy. If it was still difficult to control the prostheses, all subjects were offered at most three calibrations and chose the best one for the tasks. These calibration steps, combined with subject practice, typically lasted 20–30 minutes per controller. Additional time was provided, as needed, until each subject confirmed that they were comfortable controlling the prosthesis. Then for control tasks, able-bodied subjects used DirCon and MapCon with either 6 or 12 electrodes (backward selected). Limb-absent subjects only used 6 electrodes for DirCon and MapCon, to shorten the experiment length to prevent fatigue. All subjects used SeqCon with 2 electrodes (manually selected, as described above). The three control strategies, number of electrodes used (only varied for able-bodied subjects) and three tasks were randomized during the experiment. Subjects were blinded to the number of electrodes in use. Three trials of data were collected for each condition.

### D. Statistics

#### 1) Calibration Quality Assessment:

The RMSEs from the calibration quality assessment satisfied the normality assumption. Thus, repeated measures analysis of variance (RANOVA) and *post hoc* paired t-tests with Bonferroni correction (significance level *p* = 0.05) were used to test for RMSE differences. Prior to RANOVA, the degree of sphericity (*ε*) was used to adjust the degrees of freedom by either the method of Greenhouse-Geisser (*ε* < 0.75) or Hyunh-Feldt (0.75 < *ε* < 1). Each RANOVA assessed all possible interactions. These interactions were not significant, unless noted otherwise in the Results. When interactions were found, we proceeded to *post hoc* pair-wise comparison of all factor combinations, since the number of combinations was small.

#### 2) Task Outcomes Involving Able-Bodied Subjects (Including Comparisons Between Able-Bodied and Limb-Absent Subject Results):

We separately averaged each outcome measure (number of box-block transfers, time per clothespin transfer, and time per door open and close cycle) across the three trials per condition. Prior to each statistical test, we evaluated the normality assumption of the test data. The number of drops per trial in box-block and clothespin tasks failed the normality test, thus a non-parametric Friedman test was used to test performance differences. All other outcome measures satisfied the normality assumption. Thus, RANOVA and *post hoc* paired t-tests with Bonferroni correction were used to test performance differences. Adjustments for degrees of freedom and treatment of interactions were performed as described above.

#### 3) Task Outcomes Involving Only Limb-Absent Subjects:

When comparing performance within a task for the limb-absent subjects, our subject pool was quite heterogeneous (2 congenital and 2 traumatic limb loss; distinct remnant musculature for each; distinct past experience with myocontrol for each), thus performance differences were tested using “n-of-1” statistical analysis (i.e., separate statistical analysis for each subject). The n-of-1 approach has been used before in prosthesis control research [36] and is well suited for heterogeneous subject pools with chronic conditions [53]. Thus, we separately conducted RANOVA (after confirming data normality) and *post hoc* t-tests with Bonferroni correction for each subject, without averaging the three trials per condition. Adjustments for degrees of freedom and treatment of interactions were performed as described above.

## III. Results

### A. Calibration Quality Assessment

**Fig. 4.**
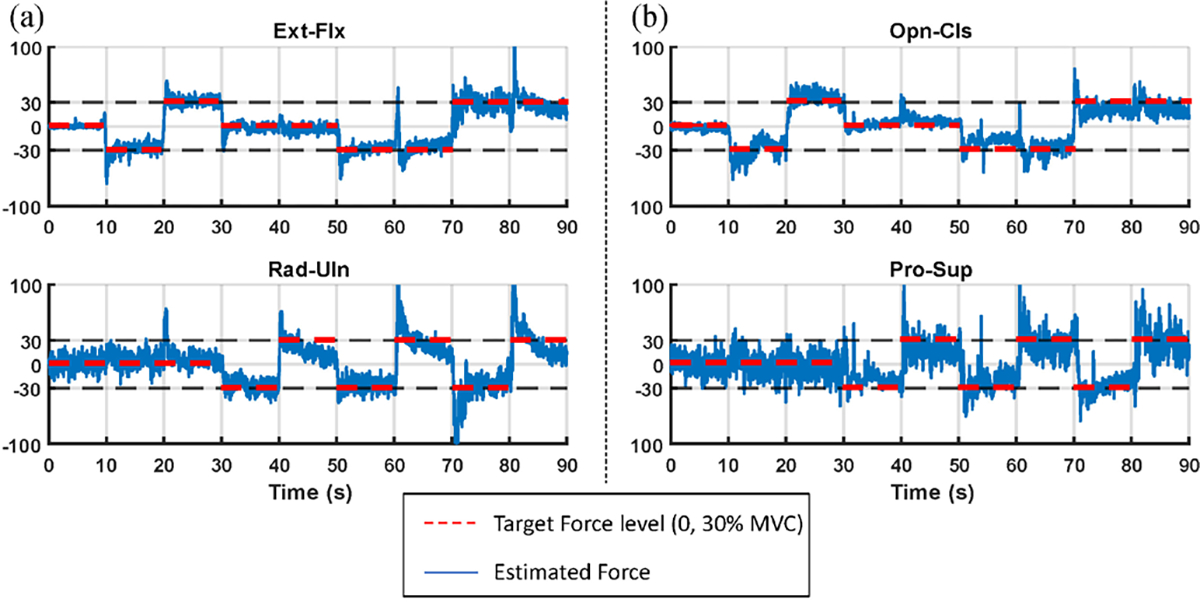
Calibration time-series examples from one subject for **a)** MapCon and **b)** DirCon. Dashed red line segments show target force level. Wavy blue lines show model-estimated force.

**Fig. 5.**
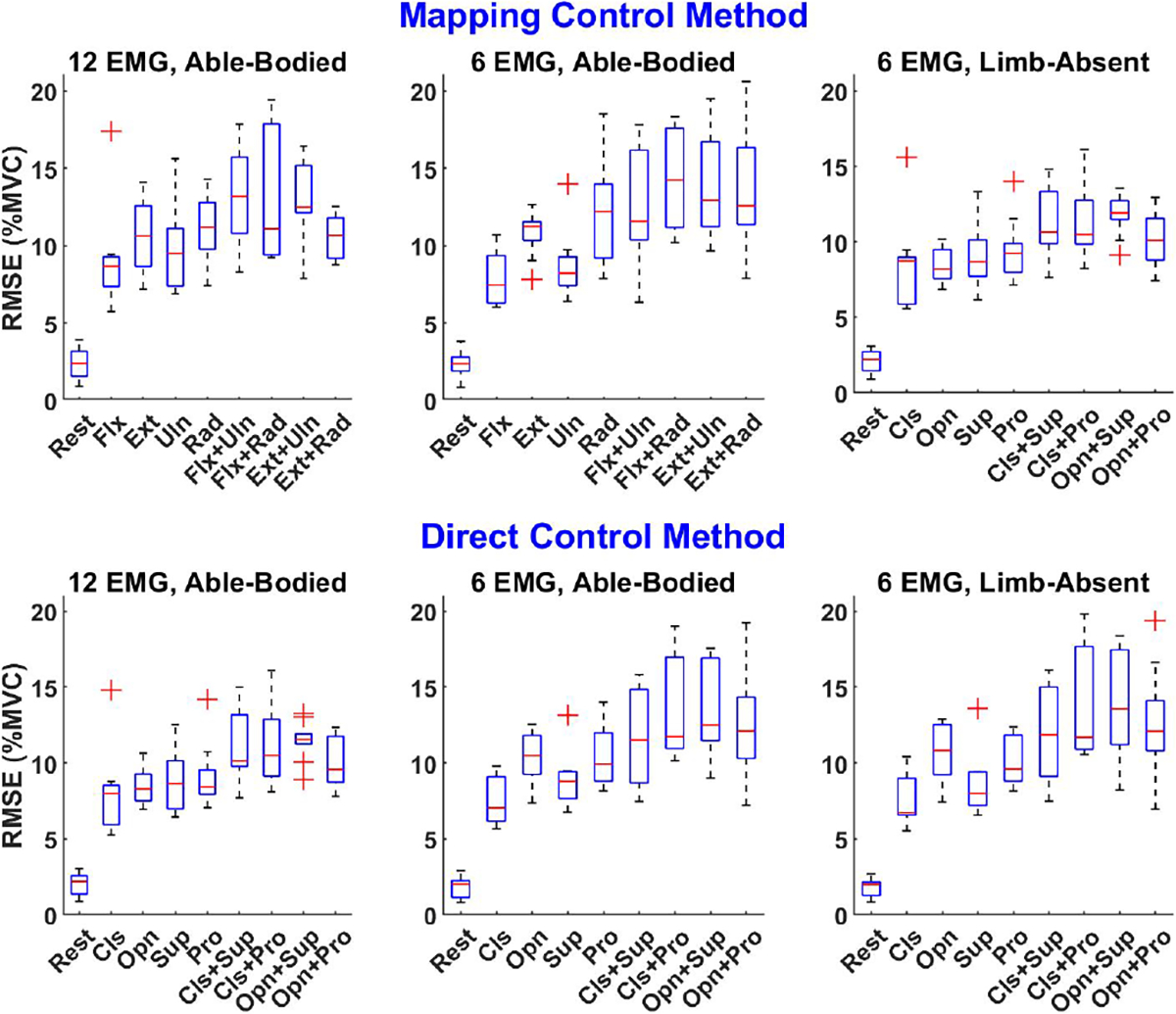
Calibration quality assessment boxplots. RMSE for each contraction type under different control methods (MapCon, DirCon) and number of EMG electrodes (6, 12) for both able-bodied and limb-absent subjects.

Fig. 4 shows example target force levels and EMG-estimated forces for a set of calibration trials. Fig. 5 summarizes across subjects the RMSE between the target %MVC and that estimated from EMG*σ* of each calibration contraction type, separately for able-bodied and limb-absent subjects, and number of electrodes retained after backward stepwise selection. Both hand and wrist errors always contributed to the RMSE, even during 1-DoF tasks. This assessment describes how well subjects can produce the desired calibration contraction, which forms the basis of the 2-DoF control algorithms.

For able-bodied subjects, a three-way RANOVA of RMSE was computed with factors: control strategy (DirCon, MapCon), number of electrodes (6, 12) and calibration contraction type (9 values, see Fig. 5). A significant interaction was found between control strategy and number of electrodes [F (1, 9) = 16.0, *p* = 0.002], while calibration contraction type was significant [F (8, 72) = 43.2, *p* < 10^−6^]. *Post hoc* comparison of the interacting factors found that for both DirCon and MapCon, 12 electrodes had lower RMSE than 6 electrodes (*p* ≤ 10^−4^). For contraction type, rest always had lower RMSE than all other types (*p* < 10^−4^), Cls / Flx exhibited lower RMSE than Pro / Rad (*p* = 0.005), Cls+Sup / Flx+Uln (*p* = 0.003), Cls+Pro / Flx+Rad (*p* = 0.006) and Opn+Sup / Ext+Rad (*p* = 10^−4^); Opn / Ext had lower RMSE than Opn+Sup / Ext+Rad (*p* = 0.004); and Sup / Uln had lower RMSE than Cls+Pro / Flx+Rad (*p* = 0.012) and Opn+Sup / Ext+Rad (*p* = 0.026).

For limb-absent subjects, a two-way RANOVA of RMS error with factors control strategy and calibration contraction type found only type was significant [*F* (1.5, 4.5) = 19.1, *p_GG_* = 0.007]. Post hoc comparison only found rest motion had lower RMSE than all others (*p* < 0.04).

### B. Box-Block Task

**Fig. 6.**
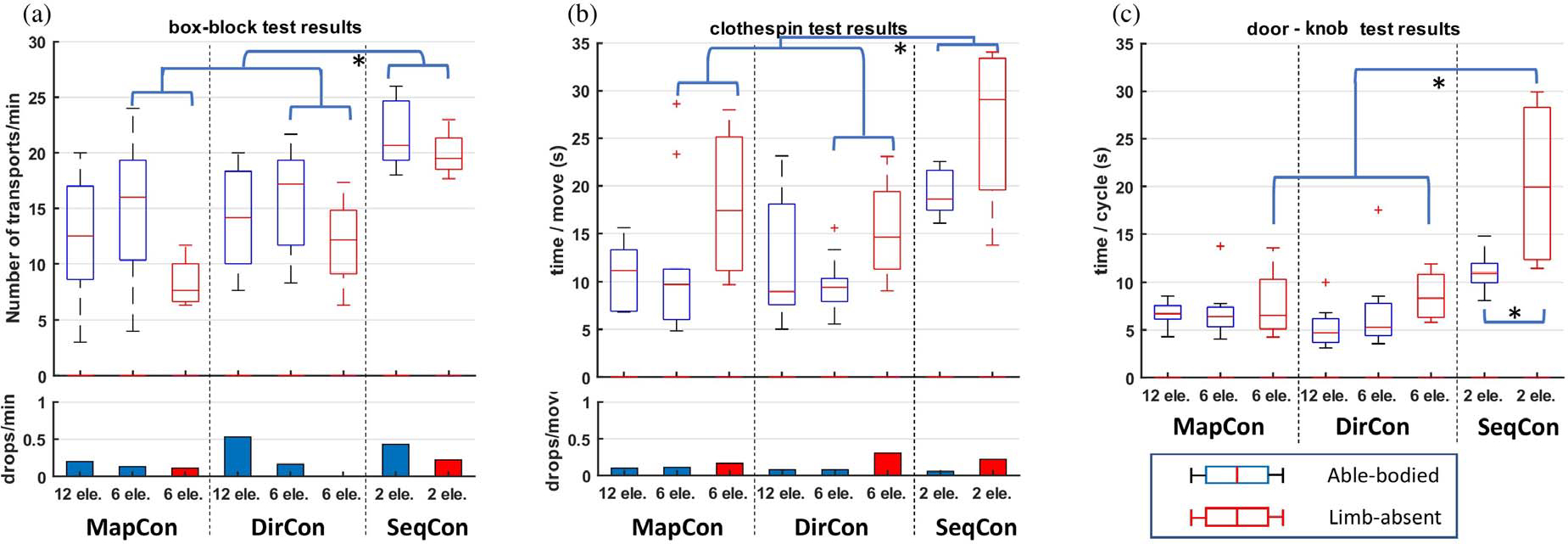
Boxplot results for **a)** box-block task (number of transfers per minute, drops per minute), **b)** clothespin task (time per move, drops per successful move), **c)** door-knob task (time per open/close cycle).

For able-bodied subjects (see Fig. 6 for summary results), the number of transfers in one minute, where more transfers represented better performance, was compared between 2-DoF control strategies (MapCon, DirCon) and number of electrodes (6, 12) via a two -way RANOVA. No statistical differences were found.

Next, we limited analysis of the 2-DoF control strategies to trials using 6 electrodes, available for both able-bodied and limb-absent subjects (see Fig. 6 for summary results). For number of transfers, a mixed two-way RANOVA with within-subjects factor of control strategy (DirCon + 6 electrodes, MapCon + 6 electrodes, SeqCon + 2 electrodes) and between-subjects factor of group (able-bodied, limb-absent) found control strategy to be statistically different [F (2, 24) = 21.62, *p* < 10^−5^], but group was not [F (1, 12) = 3.285, *p* = 0.095]. *Post hoc* comparison found that SeqCon transferred significantly more blocks than both MapCon (*p* < 10^−3^) and DirCon (p = 0.004). Note that while using SeqCon on this task, mode switching was not disabled. Nonetheless, the task was completed predominantly using only the hand DoF, and body/elbow/shoulder movement. Separately, a Friedman test on number of drops per trial (able-bodied subjects only) found no significant difference between the three control strategies.

**Fig. 7.**
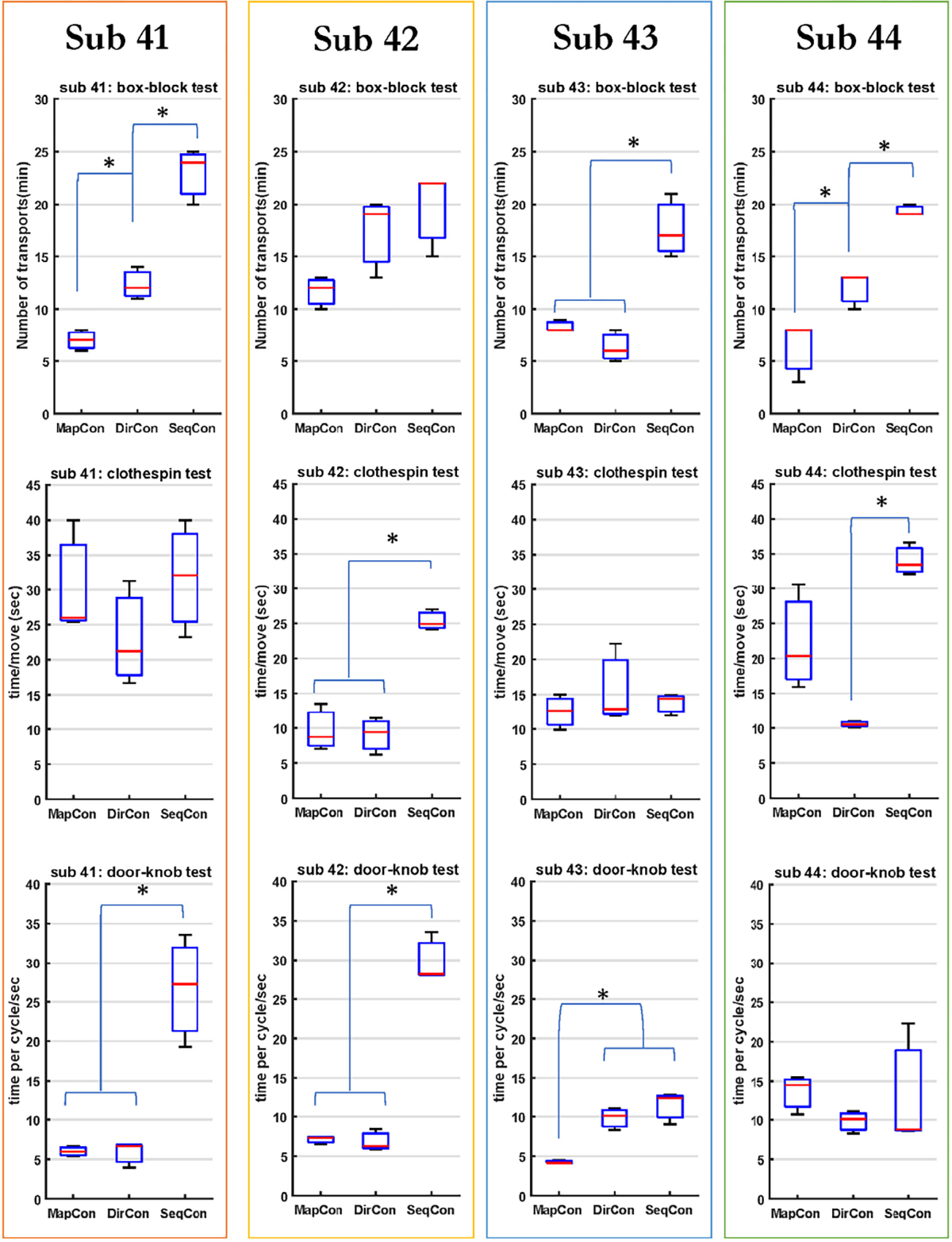
Boxplot results for each limb-absent subject for the box-block task (top row), clothespin task (middle row), and door-knob task (bottom row).

Additionally for each limb-absent subject, the number of transfers in one minute (see Fig. 7) was compared between three different control strategies (MapCon+6 electrodes, DirCon+6 electrodes, SeqCon) via a one-way RANOVA, with *post hoc* comparison made when a significant difference was found. The three trials per condition were not averaged. For three of the four subjects, the RANOVA was significant [F (2, 6) > 27, *p* ≤ 0.001], with *post hoc* comparison showing that SeqCon transferred more blocks (by a factor of 2–4) than either MapCon or DirCon (*p* < 0.038). For two of the associated *post hoc* evaluations, DirCon also transferred more blocks than MapCon (*p* < 0.038).

### C. Refined-Clothespin Relocation Task

For able-bodied subjects (Fig. 6), the time per move, where shorter time represented better performance, was compared between 2-DoF control strategies (MapCon, DirCon) and number of electrodes (6, 12) via a two-way RANOVA. No statistical differences were found.

Next, we limited analysis of the 2-DoF control strategies to trials using 6 electrodes, available for both able-bodied and limb-absent subjects (Fig. 6). For time per move, a mixed two-way RANOVA with within-subjects factor of control strategy (DirCon + 6 electrodes, MapCon + 6 electrodes, SeqCon + 2 electrodes) and between-subjects factor of group (able-bodied, limb-absent) found control strategy to be statistically different [F (1.27, 15.24) = 16.97, *p_GG_* < 10^−4^], but group was not [F (1, 12) = 0.007, *p* = 0.93]. *Post hoc* comparison found that SeqCon took significantly longer time than both MapCon (*p* = 0.003) and DirCon (*p* = 10^−5^). Separately, a Friedman test on number of drops per successful move (able-bodied subjects only) found no significant difference between the three different control strategies.

Additionally, for each limb-absent subject, time per move (Fig. 7) was compared between three different control strategies (MapCon+6 electrodes, DirCon+6 electrodes, SeqCon) via a one-way RANOVA, with post hoc comparison made when a significant difference was found. The three trials per condition were not averaged. For two of the four subjects, the RANOVA was significant [*F* (2, 6) = 11, *p* ≤ 0.009], with *post hoc* comparison in both showing that SeqCon required more time (poorer performance) than DirCon (p ≤ 0.01). For one of these subjects, SeqCon also required more time than MapCon (p = 0.001).

### D. Door-Knob Task

For able-bodied subjects (Fig. 6), the time per door-open-close cycle, where shorter time represented better performance, was compared between 2-DoF control strategies (MapCon, DirCon) and number of electrodes (6, 12) via a two -way RANOVA. No statistical differences were found.

Next, we limited analysis of the 2-DoF control strategies to trials using 6 electrodes, available for both able-bodied and limb-absent subjects. For time per cycle, a mixed two-way RANOVA with within-subjects factor of control strategy (DirCon + 6 electrodes, MapCon + 6 electrodes, SeqCon + 2 electrodes) and between-subjects factor of group (able-bodied, limb-absent) found significant interaction between these two factors [F (2, 24) = 3.8, *p_GG_* = 0.037]. We proceeded to paired *post hoc* comparisons, finding that with the SeqCon control strategy, limb-absent subjects required more time than able-bodied subjects (*p* = 0.008); and with the limb-absent subject group, SeqCon required more time than both MapCon (*p* = 0.005) and DirCon (*p* = 0.01).

Additionally, for each limb-absent subject, time per cycle was compared between three different control strategies (MapCon+6 electrodes, DirCon+6 electrodes, SeqCon) via a one-way RANOVA, with *post hoc* comparison made when a significant difference was found. The three trials per condition were not averaged. For two of the four subjects, the RANOVA was significant [F (2, 6) > 24, *p* ≤ 0.001], with *post hoc* comparison finding that SeqCon required more time than either of MapCon (*p* ≤ 0.003) or DirCon (*p* ≤ 0.003). For one other subject, the RANOVA was significant [F (2, 6) = 20.72, *p* = 0.002], with *post hoc* comparison finding that SeqCon and DirCon each required more time than MapCon (*p* ≤ 0.009).

## IV. Discussion

This research assessed the performance of regression-based 2-DoF simultaneous, independent and proportional myoelectric prosthesis control with different control strategies (DirCon, MapCon) and number of optimally-sited electrodes (6, 12), as compared to conventional sequential control (SeqCon). Evaluation was tested on standard box-block task (1-DoF assessment), refined-clothespin relocation task (2-DoF assessment) and a door-knob task (2-DoF assessment). The overall results showed no significant difference between 6 and 12 electrodes. When tested on limb-absent subjects with only 6 electrodes, all subjects successfully controlled the prostheses to complete the tasks. Both MapCon and the more intuitive DirCon exhibited good performance, indicating they could be potential approaches for 2-DoF control.

### A. Calibration Quality Assessment

In this study, subjects were offered up to three calibration trials, and could self-select the “best” trial after being given ample time to become comfortable with controlling the prosthesis. We presumed that a calibration with low EMG-force RMSE facilitates successful 2-DoF control, and vice versa. Hence, we assessed EMG-force performance of the accepted trial. The principal findings were that RMSE was lower during rest contractions and that 12 electrodes provided better EMG-force estimation than 6. The rest result is likely due to the fact that subjects can easily maintain a reproducible rest contraction, even in the absence of force feedback. But, it is difficult to accurately maintain a fixed active force level in the absence of feedback [54], [55], leading to poor tracking of the target force. One possible future solution is to feedback EMGσ in real time, which still avoids the need for measurement of force.

The finding that offline EMG-force estimation improved with 12 electrodes vs. 6 has been noted previously [28], [31]. Anecdotally, however, we found that subjects were not necessarily choosing the calibration trial with the lowest RMSE. In fact, some low RMSE calibration trials produced control models in which subjects could not actuate in one of the directions (i.e., no movement achievable). These calibrations were not selected. Nonetheless, a better metric might be the worst-case error out of the various control directions within a calibration trial, or some other metric that insures robust performance in all movement directions. This issue of strong offline EMG-force estimation not correlating to strong online prosthetic control has been noted by past studies. But, it is postulated that subjects can learn and adapt to the forward dynamics of the prosthesis in regression-based proportional control processors, perhaps reducing the requirement for highly accurate forward dynamics [19], [56]. Similarly, some studies of classification-based myocontrol of prostheses have found that high offline classification accuracy does not necessarily lead to high online performance [25], [57], [58]. These observations are disconcerting, since online performance evaluation is far more expensive and time-consuming than offline (in which many different processing schemes can be evaluated, with many parameter variations), which likely slows the advancement of control algorithms. To combat this problem, recent investigation found that a combination of offline performance metrics [59], or alternative metrics [60], better correlated with online performance in classification-based controllers. Thus, a path may still exist for classification-based offline prosthesis control algorithm development, which would be a welcomed efficiency. Perhaps similar metrics can be developed for proportional control algorithms. In any case, further investigation is warranted to develop a self-assessment of calibration quality.

We calibrated using 10 s contractions at 30% MVC effort. It is likely that shorter durations would yield similar EMG-force performance, and thus be more convenient [61], [62]. Other effort levels might also be more appropriate, and could be investigated in the future. In fact, it is not clear that the same effort level should be prescribed for each movement direction. What is most important seems to be controllability. Additional gain (or gain attenuation) could be applied to each movement direction by the controller. Further, selection of the various noise floor thresholds also could strongly influence controller performance.

### B. Sequential Control With Co-Contraction Was Better for 1-DoF Box-Block Task

Considering trials using 6 electrodes, SeqCon had (statistically significant) higher number of transports per minute on the 1-DoF box-block task than each of MapCon and DirCon. Because we didn’t lock wrist rotation during this task, 2-DoF control had the risk of unwanted wrist rotation, after which subjects lost time realigning the wrist to grasp the next block (e.g., similar to [63]). Subjects reliably contracted flexor muscles, then extensor muscles to grasp and release blocks, respectively. Therefore, an option to switch temporarily to 2-site SeqCon may be necessary within advanced prostheses controllers as an alternative scheme during activities when only Opn-Cls (1-DoF) contractions are required.

### C. Two-DoF Control Was Best for 2-DoF Tasks

Sequential control is a complicated approach for 2-DoF control. None of our able-bodied subjects had prior experience using co-contraction for mode switching, thus required a relatively long training time. One limb-absent subject had used a prosthesis with EMG co-contraction mode switching for several years, so achieved complete calibration in less than 5 minutes. The remaining limb-absent subjects struggled to learn the skill. Their imbalanced contraction between flexion and extension muscles made co-contraction difficult. EMGσ from one channel often increased faster than the other, thus the difference between the two channels caused prosthesis movement prior to triggering the desired co-contraction. We mitigated this issue by rigorous selection of thresholds, but could not completely avoid it. Furthermore, frequent co-contraction is likely to cause fatigue.

Multi-DoF control is the trend for future prostheses development. Several virtual studies utilizing classification tests [64], [65] and/or target tracking [32], [39] have shown that limb-absent subjects can control a virtual 2-dimensional movement task with high precision. Using a physical prosthesis, all our limb-absent subjects had no difficulty realizing simultaneous, independent and proportional 2-DoF control, without prior experience doing so. Some prior research has found poorer performance when using Pro-Sup inputs, perhaps due to electrode shift over muscle during Pro-Sup rotation or because key active muscles (e.g., supinator) are found deeper within the forearm and may not have EMG that is as identifiable at the skin surface. In contrast, summarizing our results across the 2-DoF tasks found that DirCon (Pro-Sup queued wrist rotation) performed similar to MapCon (Rad-Uln queued wrist rotation), and these two control strategies performed noticeably better than SeqCon.

To realize 2-DoF control, four distinct patterns/dimensions of EMG signals should be generated and then distinguished by the controller. For MapCon, which utilized more distinct wrist actions (Ext-Flx and Rad-Uln), subjects found little difficulty in separately controlling prosthesis open, close, pronate, supinate, or their combinations. But for DirCon, which utilized less distinct wrist actions (Opn-Cls and Pro-Sup), some subjects inadvertently produced wrist supination when attempting to trigger hand open. To reduce these errors, some subjects slowly opened the prosthesis hand, or triggered prosthesis hand open by simultaneously activating native/phantom hand open with low-effort pronation. We largely mitigated this problem by setting higher Sup thresholds, reducing the sensitivity of rotation. Subjects seemed to prefer this higher threshold, since they seemed to prioritize hand open/close performance, achieving small hand rotations through body posture and shoulder movement.

Another principle to realize 2-DoF control is the ability of subjects to reproduce the same EMG patterns as during calibration. For able-bodied subjects, reproducibility is facilitated by feedback from their real hand and wrist to produce the same motions. Limb-absent subjects do not have this advantage. In fact, congenital limb-absent subjects will never have experienced these feedback sensations. These differences may explain, in part, why the able-bodied subjects performed better than the limb-absent subjects on the 2-DoF door-knob task. Accordingly, congenital limb-absent subjects may be more amenable to MapCon, since they would be mapping “motions” which they have never experienced in the first place. If novel motor patterns are to be learned, selection of patterns that are more distinguishable from surface EMG are likely to be beneficial.

Traditionally, multi-DoF control is assumed to best be facilitated by selecting intuitive control strategies/phantom limb contractions [23], [66]–[68]. Indeed, limb-absent subjects have also opined this assumption [69]. However, recent evidence suggests that, with multiday training, feedback can be used to habituate non-intuitive muscle synergies that might be more advantageous for prosthesis control [70], [71]. Hence, multiday studies, which are more reflective of actual prosthesis use, may be necessary to best contrast the advantages of intuitive contractions vs. those which may be less intuitive but perhaps better for prosthesis control (after training).

### D. Number of Electrodes and Channel Selections

Six or 12 optimally-sited electrodes demonstrated no significant difference when subjects controlled the prostheses for any of the tasks, even though 12 electrodes provided better EMG-force estimation during calibration of both DirCon and MapCon. The tasks and conditions were randomized and subjects were blinded to the number of active EMG channels in use. Most subjects could recognize the difference between 12 vs. 6 channels due to different channel selection and different coefficients, but they could not tell which option provided better control. Six electrodes are reasonable to apply on a commercial prostheses considering cost, complexity and required microcontroller computation speed. When 6 electrodes were applied on limb-absent subjects, they could easily control the prosthesis after practice. Since adjacent EMG signals are highly correlated, a further increase in EMG channels introduces more redundant information, along with increased risk of electrode shorting, lift-off, etc. [72]. It is possible that even fewer than 6 electrodes might be acceptable, although not likely less than 4 for simultaneous, proportional and independent 2-DoF operation [28]–[30]. We used backward stepwise selection from 16 candidate electrodes to reduce the number of electrodes to 12 or 6. In practice, this selection step would be part of the prosthesis fitting operation completed by a prosthetist and, thereafter, the electrode sites would be fixed into their socket. Though different subjects had their own best electrode locations, the selected electrodes were always spread around the limb, not concentrated in one muscle region.

### E. Limb-Absent Subject Performance

Each limb-absent subject had prior myoelectric prosthesis control experience, completed mirror-box training before the experimental trials, and received practice time with each controller. Anecdotally, the mirror-box training was not judged by the subjects to be essential, since their prior myoelectric prosthesis use seemed to guide their perceived contraction pattern preferences. Nonetheless, we anecdotally observed that subjects became more skilled in the use of the prosthesis trial by trial. These learning effects were mitigated in our statistical comparisons because we randomized the testing order for each subject. Hahne *et al.* [36] compared 2-DoF, regression-based hand-wrist prosthesis control performance in five limb-absent subjects across two days, and found some improvement on the second day. They postulated that prosthesis control might benefit from interactive learning; the algorithm learns the EMG signal patterns from the user and generates corresponding coefficients, then the users learn how to use the prosthesis, etc.

The statistical tests using only limb-absent subject data variously found significance for the box-block, clothespin and door-knob tasks, suggesting that different subjects exhibited unique differences in performance. Numerous preexisting factors—such as muscle contraction ability, length of prosthesis use, limb-loss type and learning ability—should greatly influence task performance. Hence, prosthesis controller implementation for different users must consider their unique needs and characteristics. Of note, all limb-absent subjects used 2-DoF control for the first time in this study, and with only 20–30 minutes of practice. Yet, each limb-absent subject performed better on each 2-DoF task using each 2-DoF controller (compared to SeqCon).

### F. Two-DoF Controller Limitations and Challenges

Though each subject could complete each of the three tasks using the 2-DoF controllers, substantial challenges remain. It was obvious that the quality of calibration was essential to a subject’s performance. For some subjects, the first calibration did not result in effective prosthesis control, perhaps because these subjects may have focused more on achieving the instructed calibration contraction profile and not on contraction efforts that would be easy for them to reproduce during real tasks. For these subjects, the second or third calibration usually led to a dramatic improvement in control. A more objective measure of calibration “success” is desired to inform the user if they need to re-calibrate for better control. Assessment of overall RMSE between target force and EMG-estimated force may be dubious. Analyzing the error from each individual motion direction after calibration might better help the user gradually develop the best patterns for everyday calibration.

Another issue was unintentional movement from another DoF. We manually applied two thresholding methods to reduce the impact from unintentional movement. However, a more reproducible, automated method for threshold selection should be developed. The unintentional movement usually happened in two cases. First, it occurred when subjects had a fast change from one motion to another. In this situation, EMG in most channels would spike, producing EMGσ values much higher than normal contraction. These contractions usually triggered a correct movement of the desired DoF, but also generated unexpected movement from another DoF. Second, unintentional movement was sometimes produced when subjects used very high force levels to control the prostheses, likely due to antagonist muscle co-contraction. In both of these cases, the contraction patterns are not present in the calibration data. It is hard to completely avoid unintentional movement during control, but effective threshold selection and lower muscular efforts can reduce the sensitivity of our current approaches. In this way, users can focus on one DoF with accurate and robust control and use an additional DoF when needed.

### G. Primary Results and Contributions of This Work

The primary results and contributions of this work include:

- The work adds to the body of evidence on the successful use of regression-based EMGσ-force models for simultaneous, independent and proportional myoelectric control of 2 DoFs in a hand-wrist prosthesis. A small number of literature studies/subjects exist in which online performance has been evaluated, processing methods vary for each, and the aggregate sample size of limb-absent subjects in these studies is even smaller. Our studies with limb-absent subjects, therefore, add substantively to the literature. We have shown that our regularization method (Moore-Penrose pseudo-inverse) can provide useful online myocontrol of a physical prosthesis.
- One-DoF controllers demonstrated some advantages in 1-DoF tasks, while 2-DoF controllers performed better in 2-DoF tasks. Prosthesis control algorithms should consider providing a mechanism for users to volitionally toggle between such controllers, in order to select the best controller for the task.
- Determining optimal locations to site EMG electrodes for prosthesis control has historically been more of an art than a science [73]. We previously introduced applying several electrodes about the limb, then selecting *offline* a minimum number of optimal electrodes via backward stepwise selection in an EMGσ-force model [28], [31]. In the current work, this method was evaluated with online tasks using a physical prosthesis, with both able-bodied and limb-absent subjects. We demonstrated that *offline* EMGσ-force estimation benefited from 12 electrodes, but *online* myocontrol performed no different with 6 optimally-sited electrodes (out of 16 total). In practice, electrode site selection would be performed during prosthesis fitting and used to select permanent electrode sites. No automated methods for site selection are available in commercial devices. Our backward selection method could provide such a method.
- This work provided considerable methods detail and discussion on the pivotal role of noise threshold selection in myocontrollers. These parameters and how they are used in the prosthesis controller tend to receive far less attention. But, most muscle effort occurs at low contraction, wherein measurement noise has a disproportionate influence [74]. Future work could look at more formal methods of noise attenuation, along with automated and reproducible selection of algorithm parameters/thresholds.
- Our work found statistical differences when comparing performance within limb-absent subjects, but these differences were not uniform. Some of these distinctions may simply reflect statistical variation. But, others may be a reminder of the unique anatomical and physiologic characteristics of each prosthesis user. That is, a “one-size fits all” solution may not be best for the limb-absent population.

## V. Conclusion

This laboratory study evaluated two regression-based 2-DoF prosthesis control methods, compared with conventional co-contraction sequential control in box-block, refined-clothespin, and door-knob tasks on both able-bodied and limb-absent subjects. We found that in the box-block task that focused on 1-DoF performance, conventional SeqCon performed better than MapCon and DirCon. In 2-DoF tasks (clothespin, doorknob), both MapCon and the more intuitive DirCon performed better than SeqCon, with faster and more robust performance. Six optimally-sited electrodes (out of 16 total) had overall similar performance with 12 electrodes and are more feasible for commercial prosthesis applications. More algorithm and hardware design to improve control comfort and robustness are appropriate next steps.
